# The profile of clinical and laboratory features of Chinese VEXAS syndrome patients with hematological abnormalities: a single-center case series

**DOI:** 10.3389/fimmu.2026.1794633

**Published:** 2026-04-16

**Authors:** Xiang Ren, Min Wang, Jiali Huo, Yujiao Jia, Jigang Xiao, Neng Nie, Jinbo Huang, Jing Zhang, Xin Zhao, Liwei Fang, Guangxin Peng, Li Zhang, Fengkui Zhang, Yizhou Zheng, Jun Shi, Meili Ge

**Affiliations:** 1 State Key Laboratory of Experimental Hematology, National Clinical Research Center for Blood Diseases, Haihe Laboratory of Cell Ecosystem, Institute of Hematology and Blood Diseases Hospital, Chinese Academy of Medical Sciences & Peking Union Medical College, Tianjin, China; 2 Tianjin Institutes of Health Science, Tianjin, China; 3 Red Blood Cell Diseases Center and Regenerative Medicine Clinic, Institute of Hematology and Blood Diseases Hospital, Chinese Academy of Medical Sciences and Peking Union Medical College, Tianjin, China

**Keywords:** case series, hemato-inflammatory disorder, hematologic abnormalities, *UBA1* gene, VEXAS syndrome

## Abstract

**Objectives:**

VEXAS syndrome is a recently characterized hemato-inflammatory disorder caused by somatic mutations in the X-linked UBA1 gene in hematopoietic cells, which remains poorly characterized in Chinese populations. This study aims to address this gap.

**Methods:**

We retrospectively analyzed 4512 consecutive patients with hematologic abnormalities at a Chinese academic hospital between June 2023 and July 2024, identifying 16 male VEXAS patients (median age 61.5 years, range 30-74).

**Results:**

All patients presented with anemia and lymphopenia, with or without neutropenia and thrombocytopenia. Diagnoses included CCUS, MDS, MGUS, as well as novel phenotypes of primary myelofibrosis and a hemolysis-like disorder. Most patients (11/16, 69%) exhibited constitutional symptoms and typical autoinflammation-associated multiorgan involvement, including skin lesions, ear chondritis, pulmonary infiltration, and deep vein thrombosis, etc. Hypercellular bone marrow was commonly seen in core biopsies and 11 patients (68.8%) exhibited typical vacuoles in myeloid and erythroid progenitors. Canonical UBA1 pathogenic variants were detected in 81.3% (13/16) of patients, with p.M41V being the dominant mutation. Three infrequent variants were also identified: c.118-1G>C, p.S56P, and p.S621C. Corticosteroids and immunosuppressants commonly provided symptomatic relief, while variable hematologic responses were achieved with androgens and erythropoiesis-stimulating agents.

**Conclusions:**

As a relatively large cohort of VEXAS syndrome characterizing Chinese patients, our findings demonstrate that VEXAS should be considered in those with cytopenia, regardless of systemic symptoms or multiorgan involvement. Increased awareness among hematologists is critical to facilitate early diagnosis via UBA1 testing. This can prevent unnecessary diagnostic procedures and guide appropriate treatment, including consideration of pre-emptive stem cell transplantation.

## Highlights

• First large Chinese VEXAS syndrome cohort reveals significant underdiagnosis.
• Novel phenotypes: primary myelofibrosis with *JAK2*-V617F, and acquired hemolytic anemia, unspecified.
• Confirms *UBA1* heterogeneity and advocates for early genetic testing for unexplained cytopenia.

## 1 Introduction

VEXAS (vacuoles, E1 enzyme, X-linked, autoinflammatory, and somatic) syndrome is a newly described disorder identified in a population of patients with both autoinflammatory manifestations and hematologic abnormalities by Beck et al. in late 2020 (1).

Using a genotype-driven approach, researchers identified VEXAS as stemming from somatic UBA1 mutations in hematopoietic stem cells. Located on the X chromosome, UBA1 encodes the ubiquitin-activating enzyme E1, essential for protein degradation. Key mutations (e.g., p.Met41) generate a defective cytoplasmic isoform (UBA1c), impairing ubiquitylation and triggering innate immune hyperactivation with excessive cytokine production. This disrupts cellular homeostasis, leading to systemic inflammation, multi-organ involvement, and hematologic abnormalities—including macrocytic anemia, cytopenias, and myeloid dysplasia—often classified as MDS or MGUS (1–3). VEXAS syndrome represents a novel prototypical disease category, hemato-inflammatory disorder, bridging the diagnostic gap between overlapping rheumatologic and hematologic conditions (4, 5).

VEXAS syndrome is X-linked, predominantly affecting males, though rare female cases—mostly with Turner syndrome or acquired X monosomy—have been reported (6–9). Notably, clinical manifestations are similar between genders (10).

Since its discovery, VEXAS syndrome has attracted significant attention among hematologists, rheumatologists, and dermatologists due to its novel insights into a highly heterogeneous disease, offering both a straightforward diagnostic test and potential for improved therapies. Although increasing cases have been reported worldwide, most originate from the U.S., Europe, Japan, and Australia, with only sporadic reports from China, Mexico, and other regions (11, 12), contributing to low diagnosis rates. Notably, population studies suggest VEXAS syndrome is more prevalent than previously believed (13).

To expand the clinical and laboratory spectrum of VEXAS syndrome, we enrolled 16 patients with hematological abnormalities confirmed in our center in approximately one year to provide a comprehensive description of this Chinese cohort, with an in-depth discussion of some newly discovered VEXAS-causing variants and their novel manifestations as well. And to the best of our knowledge, this is the largest Chinese cohort of VEXAS syndrome to date.

## 2 Methods

### 2.1 Patients

We retrospectively reviewed next-generation sequencing (NGS) panel for suspected hematological diseases results from 4512 consecutive patients and identified 77 with *UBA1* variants. Those with somatic pathogenic *UBA1* variants and typical VEXAS manifestations were directly enrolled. Cases with likely pathogenic variants underwent thorough clinical review and literature validation to confirm or exclude the diagnosis. Germline mutations and female patients with normal karyotypes were excluded.

Clinical diagnosis followed the 2017 WHO classification guidelines (14, 15). The study was approved by the Institutional Review Board and Ethics Committee, complying with the Declaration of Helsinki. Written informed consent was obtained from all participants in accordance with the study protocols.

### 2.2 Acquisition of clinical inflammation and laboratory data

Clinical data were systematically collected using a customized Excel spreadsheet, incorporating electronic medical record (EMR) extracts and follow-up outcomes from telephone, WeChat, or in-person evaluations. All structured data were manually reviewed prior to analysis. Key laboratory parameters included complete blood counts, biochemical indices, inflammatory cytokines, and bone marrow morphology, pathology, cytogenetics, and molecular genetics.

### 2.3 Clinical diagnoses and definitions

Patients with typical manifestations and disease-causing variant of *UBA1* were directly diagnosed. Suspicious individuals were further evaluated based on the latest evidence. Definitions of constitutional symptoms, organ involvement, laboratory abnormalities, and rheumatological & hematologic disease entities were confirmed in accordance with previous literatures (1, 2, 8, 15, 16). The WHO 2016 revision criteria (14) for MDS and the 5th version (WHO 2022) (15) were used to diagnose and classify MDS, and International Working Group criteria were used to diagnose and classify plasma cell neoplasm (17). For patients diagnosed with MDS, the revised International Prognostic Scoring System (IPSS-R) and the molecular IPSS (IPSS-M) were calculated as previously described (18, 19).

### 2.4 Identification of *UBA1* gene variants and associated molecular abnormalities

*UBA1* was sequenced using a targeted NGS panel covering 364 genes linked to hematological disorders (**Supplementary Table 1**). DNA was extracted from bone marrow aspirates obtained at initial visit. Putative germline variants in *UBA1* (gnomAD-present with VAF > 90% in males or 40 - 60% in females) were excluded. Putative somatic variants were assessed for X chromosome copy number status. Co-mutated genes were also analyzed to delineate the molecular profile of VEXAS-associated MDS.

### 2.5 Statistical analysis

Statistics were calculated using GraphPad Prism10. Laboratory values were obtained and analyzed as continuous variables with means and SDs. Comparisons were performed using both unpaired t-test (parametric) and Mann-Whitney U test (non-parametric), with statistical significance set at *p* < 0.05.

## 3 Results

### 3.1 Identification of pathogenic variant of *UBA1* and diagnostic confirmation

**Figure 1:**
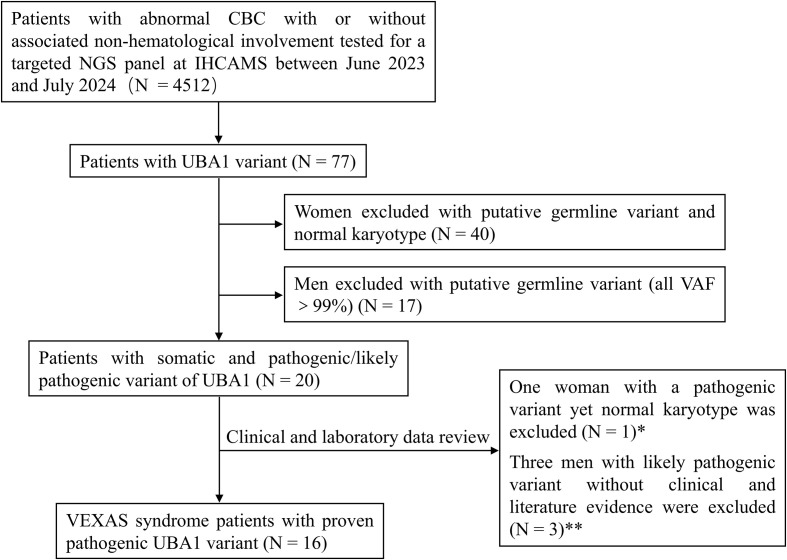
Flow chart of patient screening and inclusion. * One female patient with a *UBA1* gene mutation (p.M41Ifs7, VAF 32.40%) was excluded due to a normal karyotype and absence of autoinflammatory manifestations. ** Three patients with likely pathogenic variants (p.E353Q, p.Y618C, c.2003 + 1->T) were excluded due to the absence of multiorgan involvement and lack of literature evidence supporting a diagnosis of VEXAS syndrome. CBC, complete blood count; NGS, next generation sequencing; IHCAMS, Institute of Hematology & Blood Diseases Hospital, Chinese Academy of Medical Science.

Of the 77 patients initially identified with *UBA1* variants, 40 females with putative germline variants and normal karyotype and 17 males with germline-like variants (VAF > 99.0%) were excluded. Twenty patients carrying somatic pathogenic/likely pathogenic variants were enrolled. After excluding four due to insufficient clinical or cytogenetic support, 16 male patients were confirmed with VEXAS syndrome (**Figure 1**), yielding an approximate prevalence of 0.355% among individuals with hematological abnormalities. All pathogenic variants are listed in **Table 1**.

**Table 1:** Pathogenic somatic *UBA1* mutations and co-occurring genetic variants in 16 patients with VEXAS syndrome.

Patient	UBA1 variant	VAF %#	Co-mutations		Disease course (month)
			Pathogenic (I)	Likely pathogenic (II)	
p1	p.M41V	78.8%	No	No	1
p2	p.M41V	80.4%	No	No	2
p3	p.M41V	75.6%	No	*EZH2* p.F32L(4.10%)	3
p4	p.M41V	88.2%	*TET2* p.E294* (2.0%)	No	1
p5	p.M41V	56.1%	*NRAS* p.G12D (4%)	No	1
p6	p.M41V	79.6%	*CUX1* p.E440* (15.4%)	*KMT2D* p.R5048H(1.4%)	5
p7	p.M41L	73.5%	No	No	2
p8	p.M41L	64.5%	*U2AF1* p.Q157P (35.9%) *PAX5* p.D2H (1.2%) *TET2* p.P1894L (0.70%)	*U2AF1* p.K195R(1.4%) *CHEK2* c.1462-2A>T(48.9%) *KMT2C* p.P821L(15.8%)	1
p9	p.M41L	62.4%	*TET2* p.D1376Y (32.0%) *EZH2* p.R583* (31.10%) *ASXL1* p.Q546* (32.1%)	*EZH2* p.P132R(30.10%)	<1
p10	p.M41L	67.00%	*JAK2* p.V617F (42.6%) *EZH2* p.R690H (51.0%) *EZH2* p.W599* (43.9%) *ETNK1* p.N155S (44.9%) *ASXL1* p.S526Afs*15 (48.6%) *BRCC3* p.Q4* (9.5%) *HLA-DRB1* p.R33* (49.8%) *CUX1* p.E1137* (7.6%)	*BCL11A* p.N756K(8.5%) *ASXL1* c.1719 + 1G>A (8.0%)	1
p11	p.M41T	80.3%	No	*ASXL2* p.E703K(14.4%)	1
P12	p.M41T	86.4%	No	*HRAS* p.L6P (4.41%)	2
p13	p.M41T	78.1%	*DNMT3A* p.R882H (39.4%)	No	6
p14	p.S56F	71.1%	*U2AF1* p.R156H (1.6%) *SETBP1* p.D868N (1.0%)	*CDKN1B* p.P137Rfs*8(12.60%)	1
p15	p.S621C	84.5%	No	No	1
P16	c.118-1G>C	77.3%	*TET2* p.R1216* (1.1%)	*ZAP70* p.T380M(40.9%)	2

VAF, variant allele frequency; No, not detected.

#*UBA1* variants consistently showed the highest VAF, supporting their pathogenicity and clonal dominance.

*All denote a nonsense mutation resulting in premature termination.

Thirteen patients (81.3%) exhibited canonical variants at codon 41 (p.M41V, n = 6; p.M41L, n = 4; p.M41T, n = 3). One carried a pathogenic splice site variant (c.118-1G > C), one a missense variant at codon 56 (p.S56P), and one a noncanonical variant in adenylation domain (p.S621C). The p.S621C carrier lacked rheumatologic or dermatologic involvement but presented with hemolytic biomarkers (elevated LDH, bilirubin, reticulocytes; low haptoglobin). Extensive diagnostic workup for hemolysis, including direct antiglobulin test (Coombs test), cold agglutinin test, Ham’s test, and urinary Rous test, was negative. Peripheral blood smear showed no schistocytes, and genetic screening for hereditary hemolytic disorders (red cell membrane, enzyme, and hemoglobin-related genes) was unremarkable. This unexplained hemolytic phenotype is consistent with a recent report (20), in which three similar cases also lacked identifiable etiology. This variant was previously validated as catalytically impaired and highly suspicious for pathogenicity in a case without overt hemolysis (13).

### 3.2 Patient characteristics and atypical phenotypes of VEXAS syndrome

All 16 confirmed patients were male, with a median onset age of 61.5 years (range: 30 - 74) (**Table 2**). All patients presented with cytopenia. Anemia was present in 100% of cases, with the majority (81.3%, 13/16) exhibiting macrocytic anemia, while the remainder showed normocytic indices. Thrombocytopenia was also frequent (77.5 ± 43.4 × 10^9^/L). Neutropenia occurred in 50%, while lymphocytopenia and monocytopenia each affected 18.8%. Constitutional symptoms were common (87.5%), including malaise (75%), fever (37.5%), and weight loss (31.3%).

**Table 2:** Demographic, clinical and laboratory characteristics of 16 male patients with VEXAS syndrome associated with hematologic abnormalities.

Patient	Age at onset(y)/Dx delay (m)	Clinical manifestations (attributed to VEXAS) and tissue/organ involvement						Laboratory findings (at presentation/before start of treatment)								Clinical diagnosis		Treatment
		Constitutional symptoms	Skin	Ear/Nose/Ocular/Joint	Pulmonary	Heart & vasculature	Other**	ANC ALC mono	HB MCV Ret#	PLT	CRP ferritin	ANA ENA ANCA APA	IFE	Morphology#	Karyotype	Hematologic (WHO2016)	Rheumatologic	
P1	61y/8m	Fatigue Fever	No	Ear chondritis, Temporo-mandibular Arthralgia associated with trismus	GGO	No	Hepatomegaly (+) Splenomegaly (+) Lymphadenopathy	2.12 1.27 0.20	58 95.7 41.9	95	133 1140.7	1:1000 Neg NA NA	Neg	Hypercellularity↑ Vacuoles + Dysplasia: EM Blasts 3% MF-0	45,X,-Y	MDS-U	Ear polychondritis; Focal pterygoid myositis	Cyclosporine Danazol Thalidomide Transfusion
P2	40y/18m	No	No	Orbital & facial painful edema Wrist & ankle joint painful edema	No	No	No	3.23 1.34 0.16	100 110.1 96.7	189	76.5 730	Neg Neg NA NA	IgGλ	Hypercellularity↑↑↑ Vacuoles + Dysplasia - Blasts 1% MF-1	46,XY	MGUS	Sweet syndrome	Methylprednisolone Erythropoietin
P3	47y/30m	Fatigue	No	No	No	DVT	Intestinal ischemic necrosis IgA nephropathy IgA vasculitis	0.62 0.50 0.08	99 110.9 48.6	86	68 577.7	Neg Neg Neg Neg	NA	Normalcellularity Vacuoles + Dysplasia: GM Blasts 2% MF-0	46,XY	MDS-MLD	IgA vasculitis and nephropathy	Steroids Sirolimus Hydroxychloroquine
P4	70y/6m	Fatigue Fever Weight loss	Plantar painful nodule	Ocular pain and swollen	Interstitial changes and patchy shadows	No	Hepatomegaly (+) Splenomegaly (+) Lymphadenopathy Orchitis	9.4 1.97 0.47	68 114.0 103.0	17	78.4 548.3	1:100 RNP± Neg Neg	Neg	Hypercellularity↑↑↑ Vacuoles + Dysplasia: GM Blasts 5% MF-0	47,XY,+8	MDS-EB1	Undifferentiated connective tissue disease	Hypomethylating agents was advised; Steroid was advised Stanozolol
P5	62y/10m	Fever Weight loss	Painful nodules in face, neck and extremities	No	No	No	Hepatomegaly (+) Splenomegaly (+)	1.29 1.89 0.10	100 112.0 94.3	69	3.14 969.2	1:100 Neg NA	Neg	Hypercellularity↑↑↑ Vacuoles + Dysplastic: GEM Blasts 0.5% MF-0	46,XY,del20q	MDS-MLD	Polyarteritis nodosa	Steroids Cyclosporine Levamisole Stanozolol
P6	30y/61m	Fever	Painful erythematous plaques in extremities	No	No	No	No	1.98 2.15 0.09	104 102.0 86.7	106	17.3 202.7	1:320 Neg NA NA	NA	Hypercellularity↑↑↑ Vacuoles – Dysplasia: GEM Blasts 2% MF-0	46,XY,t(3;12)	MDS-MLD	Unspecified skin involvement	Prednisone Methotrexate
P7	66y/24m	Fatigue	Papules, nodule, pustules in face, neck, trunk and extremities	No	Infiltrate lesion	DVT	Hepatomegaly (+) Splenomegaly (++)	3.34 0.99 0.36	60 116.7 22.2	105	21.8 415.6	Neg Neg NA	Neg	Hypercellularity↑↑↑ Vacuoles + Dysplasia: GEM Blast 5% MF-1	46,XY	MDS-EB1	Sweet syndrome	Hypomethylating agents was advised; Cyclosporine Danazol Steroids
P8	68y/6m	Fatigue Weight loss	No	No	No	No	Hepatomegaly (+) Splenomegaly (+)	1.19 0.95 0.12	66 110.2 25.8	57	10.4 460.1	Neg Neg NA	NA	Hypercellularity↑ Vacuoles – Dysplasia: GEM Blasts 1% MF-1	46,XY	MDS-MLD	No	Cyclosporine+Levamisole Stanozolol
P9	61y/6m	Fatigue	No	No	No	No	No	1.93 1.38 0.18	47 93.3 9.5	42	4.18 1135.2	Neg Neg Neg Neg	Neg	Hypercellularity↑↑↑ Vacuoles – Dysplasia: M Blasts 0% MF-0	46,XY	PRCA-like MDS	No	Prednisone Cyclosporine Transfusion
P10	69y/10m	Fatigue	No	No	NA	No	No	11.78 0.88 0.16	69 86.6 7.3	50	2.41 3481	Neg Neg NA NA	NA	Normalcellularity Vacuoles – Dysplasia – Blasts 0% MF-2	46,XY	PMF	No	Luspatercept Ruxolitinib Prednisone Thalidomide Transfusion
P11	74y/7m	Fatigue	No	No	GGO, consolidation, pleural effusion	Pericardial effusion DVT	Hepatomegaly (+) Mediastinal axillary lymphadenopathy	2.97 0.83 0.20	55 112.4 28.0	139	67.8 454.2	1:100 Neg Neg Neg	IgGκ	Hypercellularity↑↑↑ Vacuoles + Dysplasia: GM Blasts 1% MF-1	46,XY	MDS-MLD MDS-MLD MGUS**	No	Erythropoietin RBC transfusion
P12	64y/14m	Fatigue Fever Weight loss	Erythematous plaques	Ear chondritis	Interstitial inflammation, localized right pulmonary atelectasis	No	Splenomegaly (+)	1.88 0.66 0.28	65 101.0 42.9	92	10.6 1224.6	1:1000 Neg Neg LA+	Neg	Hypercellularity↑ Vacuoles + Dysplasia – Blasts 0% MF-0	46,XY,t(5;12)	CCUS	Relapsing polychondritis, systemic lupus erythematosus	Methylprednisolone Cyclophosphamide Mycophenolate mofetil Cyclosporine Colchicine Tocilizumab Transfusion
P13	53y/77m	Fatigue Fever Weight loss	Papules scattered in the whole body	No	Patchy shadow	No	Inguinal lymphadenopathy	0.68 0.60 0.18	69 119.9 28.9	63	66.6 146.5	1:1000 Neg Neg NA	Neg	Hypercellularity↑↑↑ Vacuoles + Dysplasia: GM Blasts 0% MF-1	46,XY	PRCA-like MDS-MLD	Unspecified skin involvement	Prednisone
P14	46y/13m	No	No	No	No	No	No	1.11 1.51 0.15	119 100.6 142.5	49	NA 231.6	NA NA NA Neg	NA	Hypercellularity↑↑↑ Vacuoles + Dysplasia: M Blasts 2% MF-1	46,XY	MDS-SLD	No	Prednisone
P15*	49y/17m	Fatigue	No	No	No	No	Splenomegaly (+)	2.0 1.44 0.29	87 120.7 716.8	41	1.86 500.8	Neg Neg NA NA	NA	Hypercellularity↑↑↑ Vacuoles – Dysplasia: EM Blasts 0% MF-1	46,XY	CCUS with hemolytic trait	No	Unknown
P16	63y/16m	Fatigue	No	Ear & Nose	No	No	Hepatomegaly (+) Splenomegaly (++)	3.37 1.64 0.35	65 102.6 51.4	40	5.99 929.2	Neg Neg NA β2IgM+	Neg	Normalcellularity Vacuoles + Dysplasia: EM Blasts 1.5% MF-1	46,XY,del(5q)	MDS-5q	Relapsing polychondritis, antiphospholipid syndrome	Steroids Cyclosporine+levamisole Stanozolol
Total##	61.5y[30y-74y] 13.5m	87.5%(14/16)	37.5%(6/16)	31.3%(5/16)	40%(6/15)	18.8%(3/16)	/	50% 18.8% 18.8%	100% 81.3% /	75%	66.7% 81.3%	/	20% 2/10	/	37.5%	CCUS:2/16 MDS: 12/16 MGUS: 2/16	/	/

Dx, diagnosis; Constitutional symptoms = fever, fatigue, weight loss, night sweat; Skin lesions = erythematous plaques, papules, nodules, pustules. Others = Hepatomegaly, splenomegaly and lymphadenopathy were confirmed by ultrasonography. GGO, ground-glass opacity; DVT, deep venous thrombosis; ANC, absolute neutrophil count; ALC, absolute lymphocyte count; Mono, monocyte count. HB, hemoglobin; MCV, mean corpuscular volume; Ret#, absolute count of reticulocyte; PLT, platelet; CRP, C-reactive protein; ANA, Antinuclear antibody; ENA, extractable nuclear antigens; ANCA, antineutrophil cytoplasmic antibodies; APA, antiphospholipid antibodies; RNP, ribonucleoprotein; LA, Lupus anticoagulant; beta2-IgM, anti-beta2 glycoprotein; IFE, immunofixation electrophoresis; Dysplasia: granulocytic (G), megakaryocytic (M), and erythroid (E) lineages. MF: the grading of marrow fibrosis; MDS, myelodysplastic syndrome; MGUS, monoclonal gammopathy of undetermined significance; MLD, multilineage dysplasia; SLD, single lineage dysplasia; EB, excess blasts; CCUS, clonal cytopenia of unknown significance; PRCA, pure red cell aplasia; PMF, primary myelofibrosis; NA, not available; Neg, negative. # The bone marrow morphological findings were listed in order: cellularity (↑: hypercellularity; ↑↑: moderately hypercellular; ↑↑↑: highly hypercellular), vacuoles (presence/absence of cytoplasmic vacuolization), dysplasia (lineages with dysplastic cells ≥10%), blasts (percentage of blast cells), and the grading of marrow fibrosis (scored according to established criteria). CRP normal range: 0.0-8.0mg/mL; ferritin, normal range: 23.9-336.2 ng/mL. # # In the Total row, several proportions reflect the percentage of cases meeting the following thresholds: Neutrophils <2 ×10^9^/L, Lymphocytes <0.8 ×10^9^/L, Monocytes <0.12 ×10^9^/L, Hemoglobin <120 g/L, MCV >100 fL, Platelets <100 ×10^9^/L, CRP >8 mg/L, Ferritin >336.2 ng/mL.

**Figure 2:**
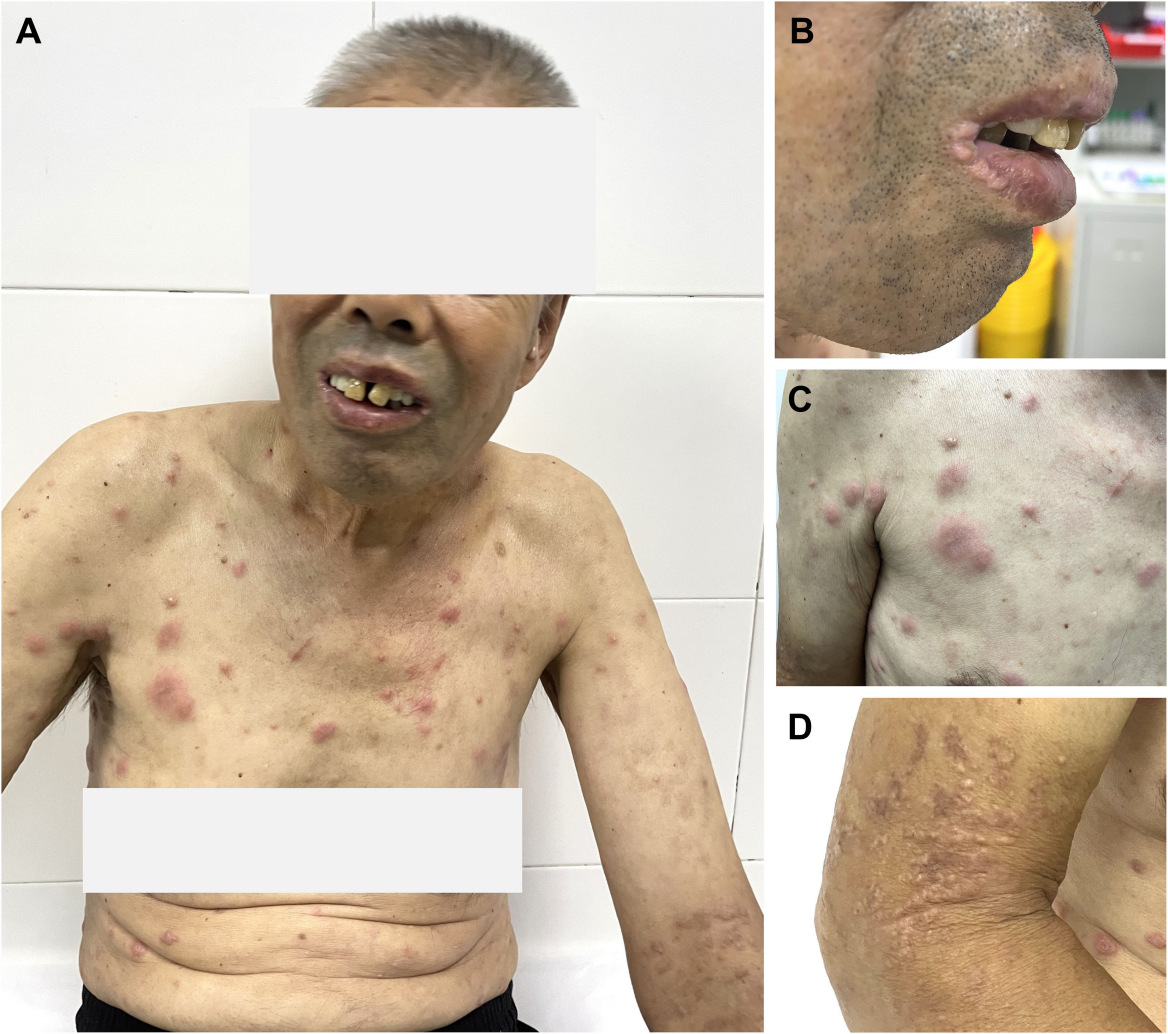
Representative cutaneous manifestations of VEXAS syndrome. One 58-year-old male patient with VEXAS syndrome presenting with infiltrated, erythematous plaques and nodules on the trunk and extremities **(A)**, accompanied by lip involvement **(B)**. The cutaneous lesions demonstrated features highly suggestive Sweet’s syndrome **(C)**, which resolved with residual light brown hyperpigmentation and scarring **(D)** (patient 7).

**Figure 3:**
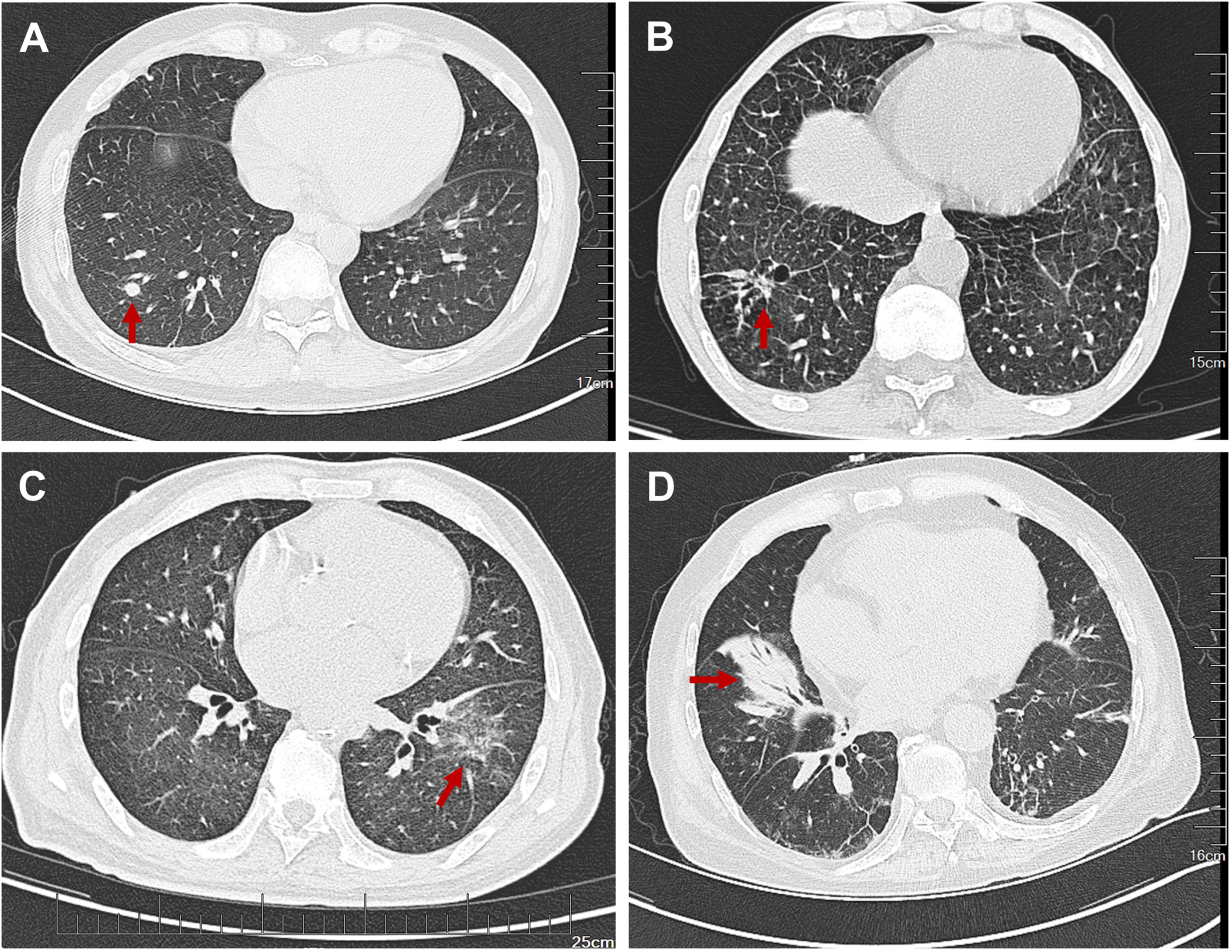
Representative CT manifestations of VEXAS syndrome patients with pulmonary involvement in this cohort. **(A)** Solid nodule with relatively smooth margins (red arrow). **(B)** Marked interlobular septal thickening with scattered ground-glass opacities and linear opacities in both lungs. A patchy consolidation and a wall-less cavity (red arrow) is visible in the right lower lobe, suggesting interstitial inflammation and emphysema. **(C)** Subtle infiltrative opacity in the left lower lobe, suggestive of infectious or inflammatory etiology. **(D)** Consolidation with atelectasis in the right lower lobe, indicative of focal lung collapse.

Multi-organ involvement was generally mild. Skin lesions (37.5%) (**Figure 2**) presented as nodules, erythema, or papules, sometimes painful; one patient (p4) had painful nodules on the soles. Ear, nose, orbital, or joint involvement occurred in 31.3%, all with typical inflammation (e.g., chondritis, periorbital swelling); two had joint symptoms, including one with temporomandibular arthralgia and trismus - a recently reported feature (21). Pulmonary abnormalities were found in 33.3% (5/15) via CT (ground-glass opacities, interstitial inflammation, effusion), though none were symptomatic (**Figure 3**). Cardiovascular involvement (deep vein thrombosis of the lower extremities) was observed in 3 patients (3/16, 18.8%). One patient (p3) was comorbid with IgA vasculitis, nephropathy, and ischemic necrosis of the intestine. Orchitis was seen only in one patient (p4).

**Figure 4:**
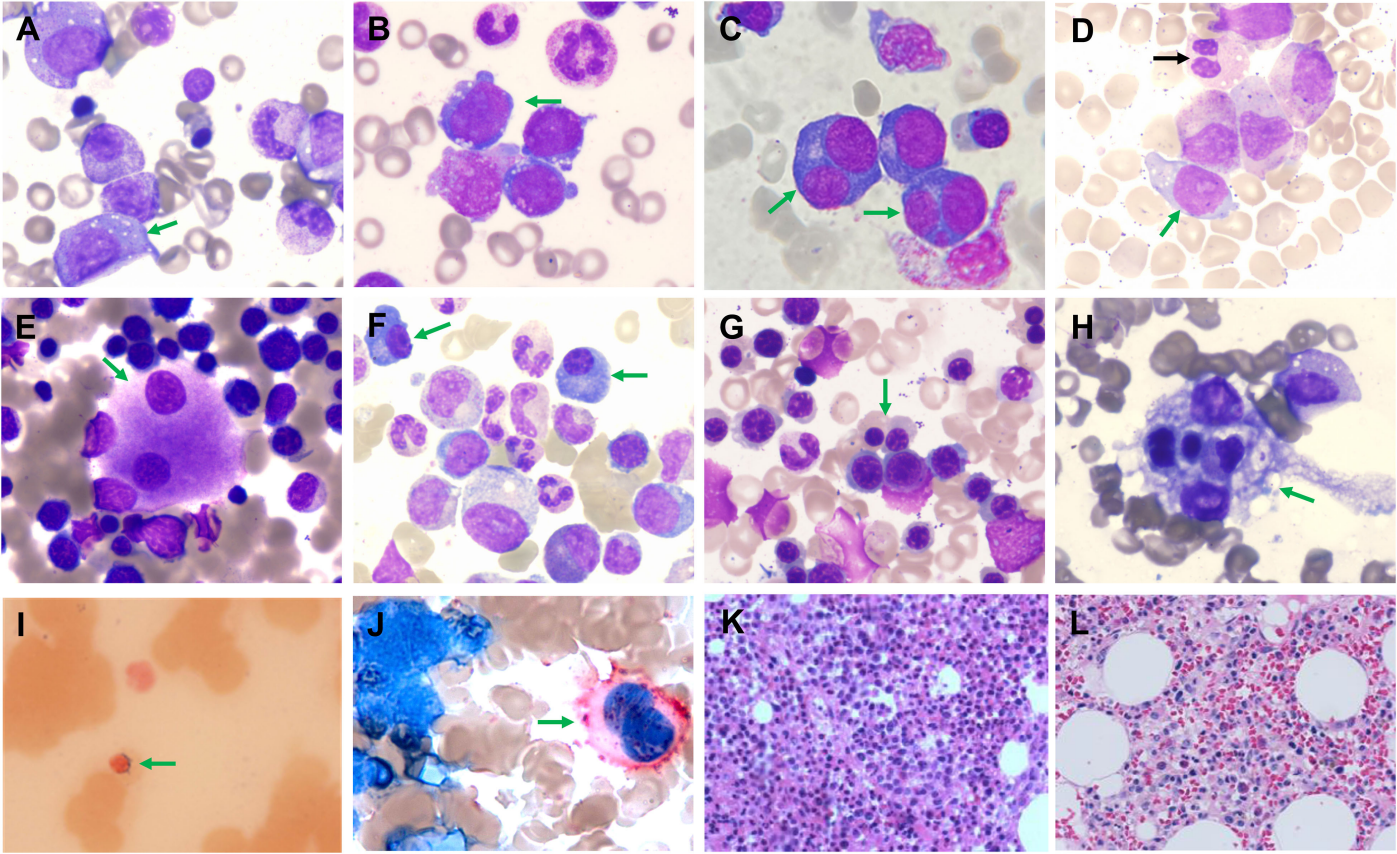
Representative morphological abnormalities found in bone marrow of VEXAS syndrome patients in this cohort. **(A)** Vacuoles in myeloid precursors (green arrow). **(B)** Vacuoles in erythroid precursors (green arrow). **(C)** Erythroid dysplasia with binucleated erythroblasts (green arrows). **(D)** Granulocytic dysplasia featuring pseudo-Pelger-Hüet anomaly (black arrow) and increased blasts (green arrow). **(E, J)** Megakaryocytic dysplasia showing binucleated megakaryocytes [**(E)**, green arrow] and CD41 immunohistochemically stained micromegakaryocytes [**(J)**, green arrow]. **(F)** Increased plasma cells (green arrows). **(G)** Hyperactive erythropoiesis in a patient with overt biochemical markers of hemolysis (green arrow). **(H)** Hemophagocytosis by macrophages (green arrow), suggesting inflammatory activation. **(I)** Ring sideroblasts (green arrow). **(K)** Hypercellular or markedly hypercellular bone marrow was observed in most patients (10/16). **(L)** Rare case of myelofibrosis (MF-2) with concurrent *JAK2* V617F mutation.

CRP was elevated in 66.7% (10/15) of cases, and ferritin in 81.3% (146.5–3335 ng/mL), with selected cases showed iron profiles consistent with anemia of chronic disease. Autoantibody profiles showed that seven patients had elevated ANA (three ≥ 1:1000), one had mild RNP positivity, and one had anti-β2 glycoprotein IgM. Several patients presented with rheumatologic conditions known to be associated with VEXAS syndrome, including relapsing polychondritis, polyarteritis nodosa, Sweet syndrome, and systemic lupus erythematosus. Whether these rheumatologic manifestations directly correlate with specific *UBA1* mutations cannot be determined from the current limited data and warrants further investigation in larger cohorts. Monoclonal immunoglobulins were detected in 2/10 patients (p2 and p11) - one with MGUS and one transiently positive for IgGκ (p11), associated with increased plasma cells (**Figure 4F**).

Bone marrow hypercellularity was seen in 81.3% in aspirate smears or core biopsies (**Figure 4K**), with 76.1% being markedly hypercellular. Myeloid hyperplasia and erythroid hypoplasia led to increased M/E ratios (68.8%), consistent with prior reports (22, 23). Two (p14/p15) showed erythroid hyperplasia and suppressed granulopoiesis (**Figure 4G**). Canonical p.M41 variants correlated with high M/E ratios, while non-canonical p.S56 and p.S621 variants were linked to low ratios. Vacuoles in myeloid and erythroid precursors were common (68.8%) (**Figures 4A, B**). Dysplasia at least one lineage occurred in 81.3%, frequently megakaryocytic (**Figures 4C–E, I, J**). Hemophagocytosis was occasionally noted (**Figure 4H**). Erythroid hypoplasia was common, with two patients showing PRCA-like morphology (p13, E = 7.5%; p9, E = 4%). Blasts were low (0 - 3%), except two with 5% blasts meeting MDS-IB1 criteria (**Figure 4D**). Most cases (12/16) were diagnosed with MDS, predominantly MDS-MLD (WHO 2016) or MDS-LB (WHO 2022), falling into low- to intermediate-risk IPSS-R categories, except one high-risk MDS-IB1 case. No MDS-IB2 or AML was observed (**Supplementary Table 2**). One patient (p10) with p.M41L variant (VAF 67.0%) was firstly observed primary myelofibrosis (MF-2) harboring a JAK2 V617F mutation (VAF 42.6%). The lower VAF of JAK2 V617F suggests it represents a secondary event.

Cytogenetic abnormalities occurred in 37.5% (6/16), including +8, -Y, del(20q), del(5q), and two rare MDS-linked translocations: t(3;12)(p21;q13) and t(5;12)(p15;q22), previously associated with VEXAS syndrome (24).

### 3.3 Circulating cytokines and lymphocyte subsets

To identify potential inflammatory mediators that may be involved in the pathogenesis of VEXAS syndrome, a panel of cytokines were measured in 7 patients. Several cytokines were elevated (**Supplementary Table 3**), though only IL-6 and IL-10 reached statistical significance when compared with healthy donors (HD) (p<0.05; **Supplementary Figure 1A**).

The proportion and absolute number of peripheral blood lymphocyte subsets was assessed by flow cytometry (**Supplementary Table 4**). The absolute counts of total lymphocytes were mostly normal in these patients and the percentages of lymphocyte subsets showed no statistical difference in VEXAS patients and HD (**Supplementary Figure 1B**). However, patients showed a significant decrease in absolute counts of all subsets (**Supplementary Figure 1C**).

### 3.4 Genetic characteristics of *UBA1* and other gene variants

As described, 13 patients with canonical *UBA1* mutations and 3 patients with recently discovered non-canonical variants were confirmed. All variants showed high VAFs (> 50%, range 56.1% - 88.2%), suggesting disease development may depend on the accumulated load of *UBA1*-mutated cells.

The screening for additional somatic mutations revealed that 12 patients (75%) had associated mutations either pathogenic (10/16) or likely pathogenic (8/16), mostly in *TET2* (n = 4), *EZH2* (n = 3), *U2AF1* (n = 2), *ASXL1* (n = 2), *CUX1* (n = 2), *JAK2* (n = 1) and *DNMT3A* (n = 1) (**Table 1**). *TET2* and *DNMT3A* were previously reported as two most common additional gene mutations in VEXAS, yet only *TET2* was dominantly detected in this cohort.

### 3.5 Interventions and outcomes

All patients exhibited anemia of varying severity, with 62.5% (10/16) requiring RBC transfusion and 6.3% (1/16) needing platelet support (**Table 2**). Glucocorticoids were administered to 68.8% (11/16) of the patients, primarily those with concurrent autoinflammatory dermatitis and chondritis. One patient (p12) with relapsing polychondritis and CCUS failed to achieve symptom remission or transfusion independence despite sequential therapies including methylprednisolone, cyclophosphamide, mycophenolate mofetil, cyclosporine, colchicine, and tocilizumab in different hospitals. For lower-risk MDS patients, treatment primarily focused on alleviating disease-related symptoms through transfusions, cyclosporine, androgens (danazol or stanozolol), erythropoiesis-stimulating agents (EPO), or luspatercept. These interventions resulted in improved hemoglobin levels or transfusion-requiring in some patients, particularly those with mild autoinflammatory manifestations. For higher-risk patients, we recommended hypomethylating agents along with early transplantation evaluation or upfront transplantation when appropriate. Of these, only two patients (p4 and p7) actually received hypomethylating agents and achieved stable disease. The variability in treatment approaches and limited follow-up highlight the need for prospective randomized trials and longer observation to establish definitive management guidelines.

## 4 Discussion

The discovery of VEXAS syndrome has elicited particular interest for clinicians and it provides a prototype of hemato-inflammatory disorder, giving the opportunity to make a precise diagnosis for patients with concomitant inflammatory and hematological abnormalities (5, 26). As the heterogeneity of clinical and laboratory manifestations of VEXAS syndrome is still not fully resolved, we here report a relatively large-scale cohort of Chinese patients to date to expand the spectrum of VEXAS syndrome.

The confirmation of 16 cases of VEXAS syndrome at one center in 13 months suggests a higher-than-expected incidence and challenges the prevailing notion of its rarity. Recent epidemiologic data from the Geisinger MyCode Community Health Initiative estimate a prevalence of approximately 1/4300 among male aged > 50 years, highlighting both the underdiagnosis and the critical need for improved clinical recognition (13, 27).

While most cases exhibited classic VEXAS features, we observed notable phenotypic variations. Although typically considered a late-onset disorder, VEXAS syndrome has been reported in patients as young as 23 years (12). A 30-year-old patient (p6) was also identified in this cohort, suggesting that younger patients with compatible symptoms should not be excluded from diagnostic consideration. Along with typical autoinflammatory features, such as ear chondritis, erythematous plaques and nodules, ocular pain and swollen, a recently reported rare manifestation of trismus secondary to focal pterygoid myositis (21) was observed in patient 1. In our cohort, the majority of patients presented with both autoinflammatory and hematologic manifestations. However, a subset of patients (6 cases in this cohort) predominantly exhibited hematologic involvement with weak or even absent inflammatory phenotypes. This indicates that VEXAS syndrome does not always present with the classic “autoinflammation + hematological abnormalities” combination; patients with isolated cytopenia should also raise suspicion for VEXAS. Recognizing this is crucial for both rheumatologists and hematologists to achieve early identification and diagnosis of VEXAS syndrome. One study has shown that patients with the *UBA1* p.S56F mutation presented exclusively with cytopenia and bone marrow failure, lacking significant inflammatory features, indicating a hematologic-predominant subtype of VEXAS (28). Furthermore, from a cytokine perspective, in contrast to previous reports (1, 25), TNF-α and IFN-γ remained unchanged in our cohort, suggesting that despite all patients having hematologic abnormalities, the autoinflammatory response in this cohort was relatively mild. Given the limited sample size (n = 7), this discrepancy should be interpreted cautiously and may reflect the distinct inflammatory status of our patients, warranting validation in larger cohorts.

We identified the first reported case of PMF (p10) with co-occurring *JAK2* V617F (VAF = 42.6%) and *UBA1* mutations (VAF = 67.0%), where the higher *UBA1* VAF suggests it may precede PMF development. Additionally, we detected a low-VAF *UBA1* variant (3.92%, p.S56F) in an unclassified MDS/MPN patient with *JAK2* V617F (27.87%), further complicating understanding of *UBA1*’s role in myeloid neoplasms. Significantly, *UBA1* consistently displayed the highest VAF among all mutated genes (**Table 1**), supporting its role as the initial driver, likely providing superior clonal expansion advantage.

Two patients in this cohort exhibited PRCA-like erythroid progenitor depletion, though its pathogenic significance remains unclear. Notably, among 116 historical VEXAS cases (8), all 12 with MGUS concurrently had MDS, suggesting shared but mechanistically undefined pathogenesis. One patient in this series has both MGUS and MDS (p11), warranting long-term monitoring. We confirmed an atypical hemolytic VEXAS subtype (initially reported by Faurel et al. (20)) in a p.S621C variant carrier (p15), highlighting the need to continuously expand the phenotypic spectrum.

Definitive diagnosis of VEXAS syndrome is established by detecting pathogenic *UBA1* variants (8, 29–31). Emerging evidence demonstrates that VEXAS syndrome exhibits a broader clinical spectrum than originally recognized (13, 20), and this expanding heterogeneity has led to proposals for a not too stringent indication to perform genetic testing (25). As NGS is routinely used in hematologic diagnostics, we strongly advocate the inclusion of *UBA1* in standard panels. And negative testing in clinically suspicious cases warrants deeper *UBA1* interrogation, including full-gene sequencing, given ongoing variant discovery.

The pathogenesis of VEXAS syndrome has been linked to loss-of-function mutations of *UBA1* in IAD and AAD domains, leading to global loss or disturbance of ubiquitination, unfolded protein response, increased cellular stress, and activation of innate immune pathways (1, 32, 33). The most frequent mutations are found at codon 41 (p.M41T/V/L), causing a decrease in the cytoplasmic UBA1b form and an increase in the dysfunctional UBA1c enzyme (1). Although these mutations affect identical genomic position, the resulting amino acid substitutions differentially impact cytoplasmic abundance of UBA1b and UBA1c. Notably, patients harboring the p.M41V variant exhibit significantly higher UBA1c production and demonstrate approximately two-fold lower cytoplasmic UBA1b levels compared to those with p.M41L/T variants. In the present study, p.M41V emerged as the most prevalent variant, though it could not be determined whether such a variant disrupts hematopoiesis more profoundly. However, it has been found that molecular variations correlate with clinical outcomes, with p.M41V demonstrating significant survival disadvantage (16). Notably, not all variants affect UBA1b expression or UBA1c accumulation. Recent work by Alyx Faurel et al. (20) found that the p.S621C variant causes a catalytic defect in the *UBA1* protein confirming, further establishing the AAD domain as a mutational hot spot.

With the increasing identification of novel pathogenic *UBA1* variants (summarized in **Supplementary Table 5**), a comprehensive assessment of the genotype-phenotype correlation is warranted. Delineating the molecular mechanisms underlying each variant may yield critical insights for therapeutic development. For instance, Ferrada et al. (16) employed isoform-specific antibodies to demonstrate different residual expression of the cytoplasmic UBA1b isoform across distinct mutations. Specifically, the p.M41T and p.M41L variants permitted some residual translation of intact UBA1b, whereas the p.M41V variant exhibited significantly reduced expression. Clinically, patients harboring p.M41V mutations presented with more severe systemic inflammatory manifestations and poorer survival, suggesting that disease severity correlates with the degree of UBA1b loss. These findings imply that residual UBA1b may be required for clonal expansion and disease pathogenesis, while near-complete loss could impair myeloid survival. Therapeutic strategies to boost residual UBA1b activity may be beneficial for VEXAS patients with variants that retain partial UBA1b expression. Promisingly, auranofin - a clinically approved rheumatoid arthritis drug - has recently been identified as a potent catalytic activity enhancer of *UBA1*, may play a role in the future (34). Additionally, molecular glue degraders (e.g., thalidomide and lenalidomide), which facilitate targeted protein ubiquitination and proteasomal degradation, may offer another mechanistic approach to modulate ubiquitination pathways in selected patients (35). Currently, VEXAS syndrome management includes diverse therapeutic approaches ranging from corticosteroids and targeted inhibitors of inflammatory signaling (such as JAK and IL-6 inhibitors) to hypomethylating agents and HSCT (36). Among these, agents that can reduce or eliminate the disease-associated clone, such as azacitidine, may represent an effective treatment option for certain VEXAS patients. Recent evidence has shown that azacitidine can reduce or clear the *UBA1* mutant clone and provide sustainable inflammatory, hematological, and molecular responses during treatment in VEXAS syndrome (37). However, standardized treatment algorithms remain undefined (38). Notably, the American College of Rheumatology has recently published the first formal international consensus guidance statement for VEXAS, developed by the International VEXAS Working Group Expert Panel (39), which will undoubtedly improve awareness, diagnosis, and management of VEXAS syndrome in the medical community. Pathogenetically, since the autoinflammatory manifestations are driven by clonal expansion of *UBA1*-mutated hematopoietic cells, we propose that targeted elimination of this aberrant clone may represent the most definitive therapeutic strategy.

VEXAS syndrome is not the first multi-system disease found to be caused by a single somatic mutation. That distinction belongs to paroxysmal nocturnal hemoglobinuria (PNH), driven by somatic *PIG-A* mutations, which stands as the historical paradigm for such disorders. Together, VEXAS and PNH collectively underscore the potential of systematic searches for somatic mutations to uncover the etiology of other multi-system diseases (37, 38).

Our study has several important limitations that should be acknowledged. First, the retrospective design and genotype-driven approach may have introduced selection bias and potential missing data. Second, the relatively small sample size may affect the statistical power and generalizability of certain findings reported here. Third, the heterogeneity of therapeutic regimens and limited treatment durations precluded robust assessment of treatment efficacy and long-term outcomes.

In summary, our findings suggest that VEXAS syndrome may be more prevalent in Chinese population than previously recognized. While clinical manifestations largely align with established phenotypes, novel hematological presentations were identified, including PMF, PRCA-like MDS, and hemolytic disorders. These findings underscore the need for heightened awareness and expertise in earlier *UBA1* detection, accurate diagnosis, and tailored management. As a bone marrow-derived disorder, VEXAS warrants longitudinal studies on *UBA1*-mutated clonal evolution and variant-specific pathogenesis. Translational research is critical to advance mechanistic therapies. As a newly defined entity, VEXAS syndrome presents unresolved questions, necessitating vigilant case identification to mitigate diagnostic oversight of atypical presentations.

## Data Availability

The data that support the findings of this study are available from the corresponding author upon reasonable request.

## Supplementary material

Supplementary Figure 1 Analysis of cytokines and lymphocyte subsets. **(A)** Comparison of cytokine levels between VEXAS patients (n = 7) and healthy controls (n = 7). IL-6 and IL-10 levels were significantly elevated in the VEXAS group (p = 0.009 and p = 0.042, respectively), indicating a more active inflammatory response. Although IL-17 showed no statistically significant difference (p = 0.57), a trend toward lower levels was observed in the patient cohort, warranting further investigation with a larger sample size. **(B)** Proportional distribution of lymphocyte subsets in VEXAS patients (n = 13) and healthy controls (n = 13). No significant differences were found between the two groups in any subset. **(C)** Absolute counts of lymphocyte subsets in VEXAS patients (n = 13) and healthy controls (n = 13). The VEXAS group exhibited significantly lower absolute counts across all lymphocyte subsets compared to healthy controls (all p-values < 0.01).

## References

[1] Beck DB Ferrada MA Sikora KA Ombrello AK Collins JC Pei W . Somatic mutations in UBA1 and severe adult-onset autoinflammatory disease. New Engl J Med. (2020) 383:2628–38. doi: 10.1056/nejmoa2026834. PMID: 33108101 PMC7847551

[2] Oganesyan A Jachiet V Chasset F Hirsch P Hage-Sleiman M Fabiani B . VEXAS syndrome: still expanding the clinical phenotype. Rheumatology. (2021) 60:e321–3. doi: 10.1093/rheumatology/keab225. PMID: 33693570

[3] Onuora S . Somatic mutations cause VEXAS syndrome. Nat Rev Rheumatol. (2021) 17:1. doi: 10.1038/s41584-020-00559-x. PMID: 33262468

[4] Gurnari C Pascale MR Vitale A Diral E Tomelleri A Galossi E . Diagnostic capabilities, clinical features, and longitudinal UBA1 clonal dynamics of a nationwide VEXAS cohort. Am J Hematol. (2024) 99:254–62. doi: 10.1002/ajh.27169. PMID: 38108611

[5] Bruno A Gurnari C Alexander T Snowden JA Greco R . Autoimmune manifestations in VEXAS: opportunities for integration and pitfalls to interpretation. J Allergy Clin Immun. (2023) 151:1204–14. doi: 10.1016/j.jaci.2023.02.017. PMID: 36948992

[6] Barba T Jamilloux Y Durel CA Bourbon E Mestrallet F Sujobert P . VEXAS syndrome in a woman. Rheumatology. (2021) 60:e402–3. doi: 10.1093/rheumatology/keab392. PMID: 33930131

[7] Stubbins RJ McGinnis E Johal B Chen LY Wilson L Cardona DO . VEXAS syndrome in a female patient with constitutional 45,X (Turner syndrome). Haematologica. (2022) 107:1011–3. doi: 10.3324/haematol.2021.280238. PMID: 34911285 PMC8968888

[8] Georgin-Lavialle S Terrier B Guedon AF Heiblig M Comont T Lazaro E . Further characterization of clinical and laboratory features in VEXAS syndrome: large-scale analysis of a multicentre case series of 116 French patients. Brit J Dermatol. (2022) 186:564–74. doi: 10.1111/bjd.20805. PMID: 34632574

[9] Sakuma M Blombery P Meggendorfer M Haferlach C Lindauer M Martens UM . Novel causative variants of VEXAS in UBA1 detected through whole genome transcriptome sequencing in a large cohort of hematological Malignancies. Leukemia. (2023) 37:1080–91. doi: 10.1038/s41375-023-01857-5. PMID: 36823397 PMC10169658

[10] Echerbault R Bourguiba R Georgin-Lavialle S Lavigne C Ravaiau C Lacombe V . Comparing clinical features between males and females with VEXAS syndrome: data from literature analysis of patient reports. Rheumatology. (2024) 63:2694–700. doi: 10.1093/rheumatology/keae123. PMID: 38407378

[11] Huang H Zhang W Cai W Liu J Wang H Qin T . VEXAS syndrome in myelodysplastic syndrome with autoimmune disorder. Exp Hematol Oncol. (2021) 10:23. doi: 10.1186/s40164-021-00217-2. PMID: 33741056 PMC7976711

[12] Sánchez-Hernández BE Calderón-Espinoza I Martín-Nares E . Challenging the paradigm: a case of early-onset VEXAS syndrome. Rheumatology. (2024) 63:e99–e100. doi: 10.1093/rheumatology/kead506, PMID: 37740251

[13] Beck DB Bodian DL Shah V Mirshahi UL Kim J Ding Y . Estimated prevalence and clinical manifestations of UBA1 variants associated with VEXAS syndrome in a clinical population. JAMA J Am Med Assoc. (2023) 329:318–24. doi: 10.1001/jama.2022.24836. PMID: 36692560 PMC10408261

[14] Arber DA Orazi A Hasserjian R Thiele J Borowitz MJ Le Beau MM . The 2016 revision to the World Health Organization classification of myeloid neoplasms and acute leukemia. Blood. (2016) 127:2391–405. doi: 10.1182/blood-2016-03-643544. PMID: 27069254

[15] Khoury JD Solary E Abla O Akkari Y Alaggio R Apperley JF . The 5th edition of the World Health Organization classification of haematolymphoid tumours: myeloid and histiocytic/dendritic neoplasms. Leukemia. (2022) 36:1703–19. doi: 10.1038/s41375-022-01613-1. PMID: 35732831 PMC9252913

[16] Ferrada MA Savic S Cardona DO Collins JC Alessi H Gutierrez-Rodrigues F . Translation of cytoplasmic UBA1 contributes to VEXAS syndrome pathogenesis. Blood. (2022) 140:1496–506. doi: 10.1182/blood.2022016985. PMID: 35793467 PMC9523373

[17] Rajkumar SV Dimopoulos MA Palumbo A Blade J Merlini G Mateos MV . International Myeloma Working Group updated criteria for the diagnosis of multiple myeloma. Lancet Oncol. (2014) 15:e538–48. doi: 10.1016/s1470-2045(14)70442-5. PMID: 25439696

[18] Greenberg PL Tuechler H Schanz J Sanz G Garcia-Manero G Solé F . Revised international prognostic scoring system for myelodysplastic syndromes. Blood. (2012) 120:2454–65. doi: 10.1182/blood-2012-03-420489. PMID: 22740453 PMC4425443

[19] Bernard E Tuechler H Greenberg PL Hasserjian RP Arango OJ Nannya Y . Molecular international prognostic scoring system for myelodysplastic syndromes. Nejm Evid. (2022) 1:EVIDoa2200008. doi: 10.1056/evidoa2200008. PMID: 38319256

[20] Faurel A Heiblig M Kosmider O Cornillon J Boudou L Guyotat D . Recurrent mutations of the active adenylation domain of UBA1 in atypical form of VEXAS syndrome. Hemasphere. (2023) 7:e868. doi: 10.1097/hs9.0000000000000868. PMID: 36999004 PMC10043588

[21] Archambeaud A Le Dreau C Bigot A Kosmider O Taleb A Boucher L . Trismus as a new feature of VEXAS syndrome. Rheumatology. (2024) 63:e258–60. doi: 10.1093/rheumatology/keae135. PMID: 38450422

[22] Patel N Dulau-Florea A Calvo KR . Characteristic bone marrow findings in patients with UBA1 somatic mutations and VEXAS syndrome. Semin Hematol. (2021) 58:204–11. doi: 10.1053/j.seminhematol.2021.10.007. PMID: 34802541

[23] Olteanu H Patnaik M Koster MJ Herrick JL Chen D He R . Comprehensive morphologic characterization of bone marrow biopsy findings in a large cohort of patients with VEXAS syndrome: a single-institution longitudinal study of 111 bone marrow samples from 52 patients. Am J Clin Pathol. (2024) 161:609–24. doi: 10.1093/ajcp/aqad186. PMID: 38413044

[24] Kanagal-Shamanna R Beck DB Calvo KR . Clonal hematopoiesis, inflammation, and hematologic Malignancy. Annu Rev Pathol-Mech. (2024) 19:479–506. doi: 10.1146/annurev-pathmechdis-051222-122724. PMID: 37832948

[25] Mascaro JM Rodriguez-Pinto I Poza G Mensa-Vilaro A Fernandez-Martin J Caminal-Montero L . Spanish cohort of VEXAS syndrome: clinical manifestations, outcome of treatments and novel evidences about UBA1 mosaicism. Ann Rheum Dis. (2023) 82:1594–605. doi: 10.1136/ard-2023-224460. PMID: 37666646 PMC10646843

[26] Diarra A Duployez N Fournier E Preudhomme C Coiteux V Magro L . Successful allogeneic hematopoietic stem cell transplantation in patients with VEXAS syndrome: a 2-center experience. Blood Adv. (2022) 6:998–1003. doi: 10.1182/bloodadvances.2021004749. PMID: 34714914 PMC8945317

[27] Tan IJ Ferrada MA Ahmad S Fike A Quinn KA Groarke EM . Skin manifestations of VEXAS syndrome and associated genotypes. JAMA Dermatol. (2024) 160:822–9. doi: 10.1001/jamadermatol.2024.1657. PMID: 38865133 PMC11170453

[28] Al-Hakim A Kulasekararaj A Norouzi M Medlock R Patrick F Cargo C . S56F UBA1 variant is associated with haematological predominant subtype of VEXAS. Brit J Haematol. (2023) 203:331–5. doi: 10.1111/bjh.19021. PMID: 37582690

[29] Poulter JA Collins JC Cargo C De Tute RM Evans P Ospina CD . Novel somatic mutations in UBA1 as a cause of VEXAS syndrome. Blood. (2021) 137:3676–81. doi: 10.1182/blood.2020010286. PMID: 33690815 PMC8462400

[30] Templé M Duroyon E Croizier C Rossignol J Huet T Friedrich C . Atypical splice-site mutations causing VEXAS syndrome. Rheumatology. (2021) 60:e435–7. doi: 10.1093/rheumatology/keab524, PMID: 34213531

[31] van der Made CI Potjewijd J Hoogstins A Willems H Kwakernaak AJ de Sevaux R . Adult-onset autoinflammation caused by somatic mutations in UBA1: a Dutch case series of patients with VEXAS. J Allergy Clin Immun. (2022) 149:432–9. doi: 10.1016/j.jaci.2021.05.014. PMID: 34048852

[32] Al-Hakim A Savic S . An update on VEXAS syndrome. Expert Rev Clin Immu. (2023) 19:203–15. doi: 10.1080/1744666x.2023.2157262. PMID: 36537591

[33] Smith JA . Regulation of cytokine production by the unfolded protein response; implications for infection and autoimmunity. Front Immunol. (2018) 9:422. doi: 10.3389/fimmu.2018.00422. PMID: 29556237 PMC5844972

[34] Yan W Zhong Y Hu X Xu T Zhang Y Kales S . Auranofin targets UBA1 and enhances UBA1 activity by facilitating ubiquitin trans-thioesterification to E2 ubiquitin-conjugating enzymes. Nat Commun. (2023) 14:4798. doi: 10.1038/s41467-023-40537-x. PMID: 37558718 PMC10412574

[35] Liu Y Bai J Li D Cang Y . Routes to molecular glue degrader discovery. Trends Biochem Sci. (2025) 50(2):134–42. doi: 10.1016/j.tibs.2024.12.006. PMID: 39753433

[36] Mekinian A Zhao LP Chevret S Desseaux K Pascal L Comont T . A phase II prospective trial of azacitidine in steroid-dependent or refractory systemic autoimmune/inflammatory disorders and VEXAS syndrome associated with MDS and CMML. Leukemia. (2022) 36:2739–42. doi: 10.1136/annrheumdis-2023-eular.127. PMID: 36104395

[37] Jachiet V Kosmider O Beydon M Hadjadj J Zhao LP Grobost V . Efficacy and safety of azacitidine for VEXAS syndrome: a large-scale retrospective study from FRENVEX. Blood. (2025) 146:1450–61. doi: 10.1182/blood.2024028133. PMID: 40373272

[38] Hadjadj J Beck DB . Improving outcomes in VEXAS syndrome: the need for prospective data. Rheumatology. (2025) 64(6):3225–6. doi: 10.1093/rheumatology/keaf041. PMID: 39862397

[39] Mekinian AM Georgin-Lavaille S Ferrada MA Savic S Koster MJ Kosmider O . American College of Rheumatology guidance statement for diagnosis and management of VEXAS developed by the International VEXAS Working Group Expert Panel. Arthritis Rheumatol. (2025) 78(3):509–22. doi: 10.1002/art.43287. PMID: 40787890 PMC12991921

