# Supplementary material for: The profile of clinical and laboratory features of Chinese VEXAS syndrome patients with hematological abnormalities: a single-center case series
Source: Front Immunol. 2026 Apr 16;17:1794633. doi: 10.3389/fimmu.2026.1794633 (PMC13128617; doi:10.3389/fimmu.2026.1794633)
Supplement: Supplementary file 2 [file DataSheet1.docx]

**Supplementary figure legend**

**Supplementary Figure S1. Analysis of cytokines and lymphocyte subsets.**(A) Comparison of cytokine levels between VEXAS patients (n=7) and healthy controls (n=7). IL-6 and IL-10 levels were significantly elevated in the VEXAS group (p=0.009 and p=0.042, respectively), indicating a more active inflammatory response. Although IL-17 showed no statistically significant difference (p=0.57), a trend toward lower levels was observed in the patient cohort, warranting further investigation with a larger sample size. (B) Proportional distribution of lymphocyte subsets in VEXAS patients (n=13) and healthy controls (n=13). No significant differences were found between the two groups in any subset. (C) Absolute counts of lymphocyte subsets in VEXAS patients (n=13) and healthy controls (n=13). The VEXAS group exhibited significantly lower absolute counts across all lymphocyte subsets compared to healthy controls (all p-values <0.01).
