# Supplementary material for: The profile of clinical and laboratory features of Chinese VEXAS syndrome patients with hematological abnormalities: a single-center case series
Source: Front Immunol. 2026 Apr 16;17:1794633. doi: 10.3389/fimmu.2026.1794633 (PMC13128617; doi:10.3389/fimmu.2026.1794633)
Supplement: Supplementary file 3 [file Table1.docx]

**Supplementary Table S1. The NGS panel encompasses 364 genes, with comprehensive analysis of all protein-coding regions**

| ABCB1 | ABL1 | ABL2 | ACTB | ACTG1 | ALDH18A1 | ANKRD26 | APC | ARID1A | ARID1B |
| --- | --- | --- | --- | --- | --- | --- | --- | --- | --- |
| ARID2 | ARID5B | ASXL1 | ASXL2 | ATG2B | ATM | ATP13A4 | ATR | ATRX | B2M |
| BACH2 | BCL10 | BCL11A | BCL2 | BCL2L1 | BCL6 | BCL7A | BCOR | BCORL1 | BIRC3 |
| BLM | BPGM | BRAF | BRCA1 | BRCA2 | BRCC3 | BRIP1 | BTG1 | BTG2 | BTK |
| CALR | CARD11 | CBL | CBLB | CBLC | CCND1 | CCND2 | CCND3 | CCR4 | CD28 |
| CD58 | CD70 | CD79A | CD79B | CD83 | CDC25C | CDKN1A | CDKN1B | CDKN2A | CDKN2B |
| CDKN2C | CEBPA | CHD2 | CHD8 | CHEK2 | CHST2 | CIITA | CLTC | CNOT3 | CNPY3 |
| CRBN | CREBBP | CRLF2 | CSF1R | CSF3R | CSMD1 | CSNK1A1 | CTCF | CUX1 | CXCR4 |
| CYLD | DAZAP1 | DDX3X | DDX41 | DIS3 | DKC1 | DNAH11 | DNM2 | DNMT3A | DNMT3B |
| DOCK8 | DTX1 | DUSP2 | DUSP22 | EBF1 | EDRF1 | EED | EGFR | EGLN1 | EGR1 |
| EIF4A2 | ELANE | EP300 | EPB41 | EPHA7 | EPOR | ETNK1 | ETS1 | ETV6 | EZH2 |
| FANCA | FANCC | FANCG | FAS | FAT1 | FAT3 | FAT4 | FBXO11 | FBXW7 | FGFR1 |
| FGFR3 | FLT3 | FOXC1 | FOXO1 | FYN | GAB2 | GATA1 | GATA2 | GATA3 | GFI1 |
| GNA13 | GNAI2 | GNAS | GNB1 | GRHPR | GSKIP | H1-2 | H1-3 | H1-4 | H1-5 |
| HAX1 | H2BC12 | HLA-A | HLA-B | HLA-C | HLA-DMB | HLA-DPB1 | HLA-DQB1 | HLA-DRB1 | HNRNPK |
| HRAS | HUWE1 | HVCN1 | ID3 | IDH1 | IDH2 | IGLL5 | IKBKB | IKZF1 | IKZF2 |
| IKZF3 | IL10RA | IL16 | IL4R | IL7R | ING1 | INO80 | IRF2BP2 | IRF4 | IRF8 |
| ITPKB | JAK1 | JAK2 | JAK3 | JUNB | KDM6A | KIT | KLF2 | KLHL6 | KLHL14 |
| KLHL21 | KLHL42 | KMT2A | KMT2B | KMT2C | KMT2D | KRAS | KRT20 | LCOR | LMO2 |
| LRRK2 | LTB | LYN | MAF | MAFB | MAP2K1 | MAP3K1 | MAP3K7 | MAP3K14 | MAPK1 |
| MAX | MBD4 | MCL1 | MECOM | MED12 | MED16 | MEF2B | MFHAS1 | MGA | MPEG1 |
| MPL | MSH2 | MTOR | MYC | MYCN | MYD88 | MYOM2 | NF1 | NFE2 | NFKBIA |
| NFKBIE | NFKBIZ | NOL9 | NOTCH1 | NOTCH2 | NOTCH3 | NOTCH4 | NPM1 | NRAS | NSD2 |
| NT5C2 | OSBPL10 | P2RY8 | PABPC1 | PALB2 | PAX5 | PDGFRA | PDGFRB | PDS5B | PHF6 |
| PIGA | PIK3CA | PIK3CD | PIK3R1 | PIM1 | PIM2 | PLCG1 | PLCG2 | POT1 | PPM1D |
| PPP1R9B | PRDM1 | PRF1 | PRKCB | PRKD2 | PRKDC | PRPF40B | PRPF8 | PRPS1 | PRRC2C |
| PSMB5 | PTEN | PTPN1 | PTPN11 | PTPRD | RAC2 | RAD21 | RASA2 | RB1 | RBBP6 |
| REL | RELN | RFTN1 | RHOA | RPL10 | RRAGC | RUNX1 | S1PR2 | SAMD9 | SAMD9L |
| SAMHD1 | SBDS | SEC24C | SETBP1 | SETD1B | SETD2 | SETDB1 | SF1 | SF3A1 | SF3B1 |
| SGK1 | SH2B3 | SH2D1A | SLC1A5 | SMARCA2 | SMARCA4 | SMC1A | SMC3 | SMO | SOCS1 |
| SP140 | SPEN | SPIB | SRP72 | SRSF2 | STAG2 | STAT1 | STAT3 | STAT5B | STAT6 |
| SUFU | SUZ12 | SYK | TAL1 | TBL1XR1 | TCF3 | TENT5C | TERC | TERT | TET1 |
| TET2 | TET3 | TMEM30A | TMSB4X | TNFAIP3 | TNFRSF14 | TNRC18 | TOX | TP53 | TP53BP1 |
| TP63 | TP73 | TPMT | TRAF2 | TRAF3 | TRIP12 | TRRAP | TYK2 | U2AF1 | U2AF2 |
| **UBA1** | UBE2A | UBR5 | USP7 | VAV1 | VHL | VMP1 | WDR24 | WEE1 | WT1 |
| XBP1 | XPO1 | ZAP70 | ZBTB33 | ZBTB7A | ZC3H12D | ZEB2 | ZFHX4 | ZFP36L1 | ZMYM3 |
| ZNF292 | ZNF318 | ZNF516 | ZRSR2 |  |  |  |  |  |  |
