# Supplementary material for: The profile of clinical and laboratory features of Chinese VEXAS syndrome patients with hematological abnormalities: a single-center case series
Source: Front Immunol. 2026 Apr 16;17:1794633. doi: 10.3389/fimmu.2026.1794633 (PMC13128617; doi:10.3389/fimmu.2026.1794633)
Supplement: Supplementary file 4 [file Table2.docx]

**Supplementary Table S2. Diagnosis and Risk Stratification of Hematological Disorders in 16 patients with VEXAS Syndrome**

| **Patient** | **Diagnosis** | | **Risk Stratification** | |
| --- | --- | --- | --- | --- |
|  | **WHO 2016** | **WHO2022** | **IPSS-R Score** | **IPSS-M Score** |
| p1 | MDS-U | MDS-LB | 3, Low | -0.28, Moderate Low |
| p2 | MGUS，ACD | / | / | / |
| p3 | MDS-MLD | MDS-LB | 4, Intermediate | 0.22, Moderate High |
| p4 | MDS-EB1 | MDS-IB1 | 7, Very High | 1.03, High |
| p5 | MDS-MLD | MDS-LB | 3, Low | -0.09, Moderate Low |
| p6 | MDS-MLD | MDS-LB | 3.5, Intermediate | -0.49, Moderate Low |
| p7 | MDS-EB1 | MDS-IB1 | 4.5, Intermediate | 0.15, Moderate High |
| p8 | MDS-MLD | MDS-LB | 3, Low | 0.13, Moderate High |
| p9 | PRCA-like MDS-SLD | MDS-LB | 3.5, Intermediate | 1.33, High |
| p10 | PMF | / | * | / |
| p11 | MDS-MLD，MGUS** | MDS-LB | 3, Low | -0.13, Moderate Low |
| p12 | CCUS*** | / | / | / |
| p13 | PRCA-like MDS-MLD | MDS-LB | 3.5, Intermediate | -0.02, Moderate Low |
| p14 | MDS-SLD | MDS-LB | 2, Low | -0.27, Moderate Low |
| p15 | CCUS**** | / | / | / |
| p16 | MDS-5q | MDS-5q | 3.5, Intermediate | 0.43, Moderate High |

*DIPSS: 3, intermediate-2 risk；DIPSS-Plus: 4, high risk；MIPSS: 7, high risk；GIPSS: 1, intermediate risk

** associated with transient detection of monoclonal immunoglobulin (IgGκ)

*** associated with 8% of ring sideroblasts

**** associated with hemolytic trait (significantly increased reticulocytes, LDH, indirect bilirubin, free hemoglobin, and decreased haptoglobin)
