# Supplementary material for: The profile of clinical and laboratory features of Chinese VEXAS syndrome patients with hematological abnormalities: a single-center case series
Source: Front Immunol. 2026 Apr 16;17:1794633. doi: 10.3389/fimmu.2026.1794633 (PMC13128617; doi:10.3389/fimmu.2026.1794633)
Supplement: Supplementary file 5 [file Table3.docx]

**Supplementary Table S3. Serum Cytokine Levels in 7 Patients with VEXAS Syndrome**

| **Patient** | **Serum Inflammatory Cytokines (pg/mL)** | | | | | | | | | | | | |
| --- | --- | --- | --- | --- | --- | --- | --- | --- | --- | --- | --- | --- | --- |
|  | | **IL-1β** | **IL-2** | **IL-4** | **IL-5** | **IL-6** | **IL-8** | **IL-10** | **IL-12P70** | **IL-17** | **IFN-α** | **IFN-γ** | **TNF-α** |
| **p4** | | 3.35 | 3.17 | **3.88** | **4.63** | **25.77** | 8.68 | **9.76** | **4.19** | 4.29 | 3.89 | 4.12 | 2.95 |
| **p6** | | 3.65 | 3.62 | **3.17** | **4.10** | **16.84** | 8.24 | **8.08** | 0.92 | 0.01 | 3.32 | 3.28 | 2.38 |
| **p7** | | 4.0 | 3.99 | **4.91** | **4.10** | **40.09** | 14.44 | **17.73** | 2.74 | 0.01 | 3.97 | 3.94 | 3.62 |
| **p9** | | 4.51 | 3.82 | **4.56** | **3.77** | 3.50 | 4.34 | **9.50** | **5.82** | 0.01 | 4.64 | 4.54 | 4.42 |
| **p11** | | 0.29 | 0.34 | 0.27 | 0.06 | **26.55** | 6.40 | **5.14** | 0.72 | 0.01 | 0.49 | 0.47 | 0.40 |
| **p12** | | 0.15 | 1.51 | 1.07 | 0.09 | 1.86 | 13.15 | **5.35** | 1.02 | 6.78 | 0.52 | 0.84 | 0.89 |
| **p13** | | 4.86 | 4.07 | **4.64** | **4.42** | **9.37** | 7.61 | **8.03** | **4.92** | 0.01 | 3.86 | 4.04 | 4.21 |
| **Mean ± SD** | | **-** | **-** | **3.21±1.85** | **3.02±2.03** | **17.71±13.95** | **-** | **9.08±4.22** | **2.90±2.10** | **-** | **-** | **-** | **-** |

Normal range: IL-1β ≤ 12.4 pg/mL; IL-2 ≤ 5.71 pg/mL; IL-4 ≤ 3.0 pg/mL; IL-5 ≤ 3.1 pg/mL; IL-6 ≤ 5.3 pg/mL; IL-8 ≤ 20.6 pg/mL; IL-10 ≤ 4.91 pg/mL; IL-12P70 ≤ 3.4 pg/mL; IL-17 ≤ 20.6 pg/mL; IFN-α ≤ 8.5 pg/mL; IFN-γ ≤ 7.42 pg/mL; TNF-α ≤ 4.6 pg/mL
