# Supplementary material for: The profile of clinical and laboratory features of Chinese VEXAS syndrome patients with hematological abnormalities: a single-center case series
Source: Front Immunol. 2026 Apr 16;17:1794633. doi: 10.3389/fimmu.2026.1794633 (PMC13128617; doi:10.3389/fimmu.2026.1794633)
Supplement: Supplementary file 6 [file Table4.docx]

**Supplementary Table S4. Lymphocyte subsets (absolute numbers and percentages) on admission (*)**

| **Ref**  **Patient** | **Total** | **CD3+ (%)** | **CD3+CD4+ (%)** | **CD3+CD8+ (%)** | **NK (%)** | **CD19+ (%)** | **CD3+ (#)** | **CD3+CD4+ (#)** | **CD3+CD8+ (#)** | **NK (#)** | **CD19+ (#)** |
| --- | --- | --- | --- | --- | --- | --- | --- | --- | --- | --- | --- |
|  | **0.8-4** | **57-88** | **27-58** | **16-45** | **3-32** | **4-18** | **466-1992** | **267-1257** | **147-778** | **51-574** | **41-374** |
| p2 | 1.34 | 71 | 37 | 29 | 9 | **20↑** | 1024 | 539 | 422 | 132 | 258 |
| P3 | **0.50↓** | 75 | 51 | 22 | 21 | 4 | **293↓** | **199↓** | **86↓** | 82 | **18↓** |
| P4 | 1.97 | 87 | 42 | 44 | 11 | **2↓** | 1393 | 668 | 707 | 175 | **35↓** |
| P5 | 1.89 | 85 | 29 | 25 | 4 | 11 | 770 | **263↓** | 224 | **32↓** | 102 |
| P6 | 2.15 | 76 | 42 | 32 | 18 | 6 | 1467 | 798 | 608 | 341 | 112 |
| P7 | 0.99 | 71 | 54 | **15↓** | 17 | 11 | 489 | 368 | **103↓** | 118 | 73 |
| P8 | 0.95 | 62 | 54 | **7↓** | **35↑** | **3↓** | **454↓** | 397 | **52↓** | 256 | **22↓** |
| P9 | 1.38 | 71 | 40 | 29 | 17 | 12 | 1008 | 567 | 418 | 241 | 167 |
| P10 | 0.88 | 64 | 37 | 26 | 16 | **20↑** | **289↓** | **169↓** | **119↓** | 72 | 91 |
| P11 | 0.83 | 90 | 50 | 37 | 5 | 4 | 1087 | 599 | 450 | 58 | 54 |
| P12 | **0.66↓** | 71 | 49 | 22 | 7 | **21↑** | **405↓** | 280 | **126↓** | **42↓** | 122 |
| P13 | **0.60↓** | 81 | 46 | 32 | 14 | 5 | **427↓** | **244↓** | 170 | 72 | **28↓** |
| P15 | 1.44 | 82 | 52 | 25 | 4 | 13 | 1134 | 717 | 339 | 51 | 184 |
| Mean ± SD | 1.20**±**0.55 | 75.85±8.66 | 44.85±7.72 | 26.54±9.34 | 13.7±8.6 | 10.2±6.8 | 787.7±419.6 | 446.8±212.4 | 294.2±213.1 | 128.6±96.9 | 97.4±72.0 |

Ref, reference range; Total, total number of lymphocytes. CD, cluster of differentiation; NK, natural killer cell (CD3-CD16+/CD56+).

Absolute numbers (#) are expressed as cells/µL.

***** The table summarizes lymphocyte subset values of 13 patients. As no consistent trends were observed, statistical analysis (e.g., mean/median) was not performed. Abnormal values (elevated or reduced) are marked in the table for individual cases. Whether the decreased lymphocyte subsets represent cells with UBA1 mutations remains unclear. Additionally, the data roughly suggest that lymphocytosis or subset expansion is uncommon in VEXAS syndrome. The observed lymphocytopenia may reflect impaired immune surveillance, potentially creating a permissive microenvironment for the expansion of UBA1-mutated clones.
