# Supplementary material for: The profile of clinical and laboratory features of Chinese VEXAS syndrome patients with hematological abnormalities: a single-center case series
Source: Front Immunol. 2026 Apr 16;17:1794633. doi: 10.3389/fimmu.2026.1794633 (PMC13128617; doi:10.3389/fimmu.2026.1794633)
Supplement: Supplementary file 7 [file Table5.docx]

**Supplementary Table S5. Currently Known Disease-causing UBA1 Variants (So Far Reported)**

| **Nucleotide Change** | **Exon/Splice Site** | **Amino Acid Alteration** | **Functional Consequence** | **Supporting Evidence** |
| --- | --- | --- | --- | --- |
| c.122T>C | exon3 | M41T | Teduced translation of the cytoplasmic isoform UBA1b and result in expression of a catalytically impaired isoform UBA1c | New Engl J Med. 2020;383:2628-38 |
| c.121A>G | exon3 | M41V |  |  |
| c.121A>C/T | exon3 | M41L |  |  |
| c.163T>C | Exon3 | Y55H | Temperature sensitive partial loss of function of UBA1 by most prominently affecting ubiquitin transfer to E2 enzymes. | EMBO J. 2024 May;43(10):1919-1946 |
| c.167C>T | exon3 | S56F(P*) | Resulted in a temperature-dependent impairment in UBA1 catalytic activity. | Blood. 2021;137:3676–81  J Clin Immunol. 2022;42:947–51  Leukemia (2023) 37:1080–1091  JAMA. 2023;329(4):318-324 |
| c.1430G>C | exon11 | G477A | Decreased E1 ubiquitin thioester formation and E2 enzyme charging, reductions in ubiquitin adenylation and thiolation. | Arthritis Rheumatol. 2023 Jul;75(7):1285-1290 |
| c.1432G>T | Exon11 | A478S | Loss of ubiquitin transfer to the E2 enzyme and reduce UBA1 activity. | EMBO J. 2024 May;43(10):1919-1946 |
| c.1516G>A | Exon 11 | p.D506N | Reductions in ubiquitin thiolation and reduce UBA1 activity. | EMBO J. 2024 May;43(10):1919-1946 |
| c.1517A>G | Exon11 | p.D506G |  | EMBO J. 2024 May;43(10):1919-1946 |
| c.1861A>T | exon16 | S621C | Loss of ubiquitin transfer to the E2 enzyme and reduce UBA1 activity | JAMA. 2023;329(4):318-324  Hemasphere. 2023 Mar 24;7(4):e868. |
| c.118-1G>C  c.118-2A>C  c.118-3T>A | Splicing site of exon3 | / | Reduction in properly spliced transcript and the creation of multiple incorrectly spliced products. | Blood (2021) 137 (26): 3676–3681.  Leukemia (2023) 37:1080–1091  JAMA. 2023;329(4):318-324  Rheumatology, 2024, 00, 1–6 |
| c.118-9_118-2del | Splicing site of exon3 | / | Affecting the splice acceptor site and resulting in the loss of translation start site of M41. | Rheumatology 2021;60:e435–e437 |
| c.119-1G>C | Splicing site of exon3 | / |  | Blood (2021) 137 (26): 3682–3684. |
| c.346-2A>G | Splicing site of exon4 | / | Results in two novel mRNA transcripts and two isoforms of UBA1 protein that are estimated to have decreased E1 enzyme activity | Rheumatology, 2024, 00, 1–6 |

*Phenylalanine is represented as F (standard) or P (historical); S56F and S56P indicates equivalent amino acid alterations in literature.
